# Hypernatremia During Intravenous Treatment With Fosfomycin: A Retrospective Medical Record Review Study and an Analysis of Spontaneous Reports in the EudraVigilance Database

**DOI:** 10.3389/fphar.2022.844122

**Published:** 2022-03-29

**Authors:** Cristina Scavone, Annamaria Mascolo, Francesca Futura Bernardi, Maria Luisa Aiezza, Paola Saturnino, Gaia Morra, Margherita Simonelli, Marida Massa, Andrea Pomicino, Giuseppina Minei, Raffaella Pisapia, Micaela Spatarella, Ugo Trama, Gaspare Guglielmi, Annalisa Capuano, Alessandro Perrella

**Affiliations:** ^1^ Campania Regional Centre for Pharmacovigilance and Pharmacoepidemiology, Naples, Italy; ^2^ Department of Experimental Medicine—Section of Pharmacology “L. Donatelli”, University of Campania “Luigi Vanvitelli”, Naples, Italy; ^3^ Regional Direction for Health Management, Pharmaceutical Unit, Naples, Italy; ^4^ Hospital Pharmacy—AORN A. Cardarelli, Naples, Italy; ^5^ Unit Emerging Infectious Disease, Ospedali dei Colli, Naples, Italy; ^6^ Hospital Pharmacy - Pharmacovigilance Unit, Ospedali dei Colli, Naples, Italy

**Keywords:** ADR, EudraVigilance, hypernatremia, intravenous fosfomycin, retrospective study, safety

## Abstract

**Background:** Hypernatremia is a serious event that can occur during intravenous (IV) treatment with fosfomycin, and it can also be caused by a wrong drug preparation. Considering the clinical significance of hypernatremia, we decided to carry out two studies by using two different data sources with the aim to evaluate cases of IV fosfomycin-induced hypernatremia.

**Methods:** A retrospective medical record review was performed from June 2017 to June 2019 using data from two hospitals in Southern Italy. The information collected was related to the patients, the antibiotic treatment regimen, type of adverse drug reaction (ADR), hypernatremia severity classification, and drug withdrawal due to ADRs. Moreover, a pharmacovigilance study was performed from the date of the European marketing authorization of fosfomycin to October 11, 2021, using data reported on the European website of suspected ADRs. Information related to the patient characteristics, treatment, hypernatremia, and type of reporter was retrieved.

**Results:** From the retrospective medical record review, a total of 62 patients (48 men and 14 women) in treatment with fosfomycin were identified, of which 17 experienced ADRs. Specifically, 11 patients experienced hypernatremia. During the period from June 2017 to June 2018, a total of 63.7% of hypernatremia events were related to the wrong reconstitution of the drug. According to these results, a surveillance and training campaign about the correct drug reconstitution was managed. However, from June 2018 to June 2019, we still had four new hypernatremia cases. Drug withdrawal occurred in only one patient with hypernatremia. From the pharmacovigilance study, a total of 25 cases of IV fosfomycin-induced hypernatremia were retrieved. No substantial difference was found for patients’ distribution by sex. Most cases were classified as serious (68%) and reported “Recovered/resolved” as the outcome (44%). In the majority of cases, fosfomycin was the only suspected drug reported (72%).

**Conclusion:** Our results show that training campaigns on the correct drug preparation need to be strengthened to allow a reduction of hypernatremia cases. Moreover, when close monitoring and management is performed by the infectious disease (ID) specialist and hospital pharmacist, there also is a reduction in antibiotic withdrawal due to hypernatremia.

## Introduction

Fosfomycin is a bactericidal antibiotic agent able to inhibit an enzyme-catalyzed reaction in the first step of the synthesis of the bacterial cell wall. Its intravenous (IV) formulation, fosfomycin disodium, is currently being used in several clinical conditions with positive results in terms of efficacy and safety (Shorr et al., 2017). However, this IV formulation can be associated with different adverse drug reactions (ADRs) that include angioedema, aplastic anemia, cholestatic jaundice, and hepatic necrosis (Michalopoulos et al., 2011; Pyrpasopoulou et al., 2018). In addition, the IV formulation is associated with a high sodium intake which represents a life-threatening clinical condition and is a limitation, especially for patients with heart failure or those on hemodialysis (Iarikov et al., 2015; Putensen et al., 2019). Hypernatremia, defined as a serum sodium level >145 mmol/L, is relatively common among the elderly and critically ill patients. Its frequency among hospitalized patients ranges from 0.3 to 3.5% (Aiyagari et al., 2006; Salahudeen et al., 2013; Thongprayoon et al., 2020). If this condition is not promptly recognized and treated, clinical consequences can be irreversible, leading to cell and organ damage and high mortality (Qian, 2019).

Considering the clinical significance of hypernatremia and given that the real frequency of IV fosfomycin-induced hypernatremia in clinical practice is still not well defined (being mostly known only in patients in intensive care units—ICUs) (Putensen et al., 2019), we carried out a pharmacovigilance study through the analysis of data collected in the European spontaneous reporting system (the EudraVigilance database) with the aim to assess the amount of Individual Case Safety Reports (ICSRs) reporting cases of fosfomycin-induced hypernatremia on a European level. In addition, we carried out a retrospective medical records review study with the aim to identify cases of fosfomycin-induced hypernatremia among patients who had received the drug in two hospitals in the south of Italy. Moreover, we used data from the retrospective medical records review to secondarily assess the impact of a regional training campaign related to the correct drug preparation on the reduction of fosfomycin-induced hypernatremia cases caused by reconstitution errors.

## Methods

### Descriptive Analysis of Data Reported in the EudraVigilance Database

Data on ICSRs with fosfomycin as the suspected drug were retrieved from the website of suspected ADRs (www.adrreports.eu) of the European pharmacovigilance database (EudraVigilance, EV). The EV is managed by the European Medicines Agency (EMA). The EV contains all ICSRs reported by a healthcare professional or a non-healthcare professional to a European Union national competent authority or a marketing authorization holder. These data are publicly available for transparency through the EMA website (www.adrreports.eu). From this website, a search of ICSRs related to fosfomycin was performed, and by using the line listing function, ICSRs reporting fosfomycin as the suspected drug and hypernatremia were retrieved from the date of marketing authorization granted by the EMA to 11 October 2021.

Information on patient characteristics (patient’s age group and sex), hypernatremia (outcome and seriousness), primary source qualification, number of suspected drugs other than fosfomycin, and number of concomitant drugs were provided for all ICSRs. In accordance with the International Council on Harmonization E2D guidelines, we classified an ICSRs as serious if it was life-threatening, resulted in death, caused/prolonged hospitalization or disability, determined a congenital anomaly/birth defect or other medically important condition. The outcome of hypernatremia was classified as “Recovered/resolved,” “Recovering/resolving,” “Recovered/resolved with sequelae,” “Not recovered/not resolved,” “Fatal,” and “Unknown.”

The reporting odds ratio (ROR), its’ 95% confidence interval (95%CI), and the chi-square test were computed to assess the reporting frequency of hypernatremia with fosfomycin compared to gentamicin.

### Retrospective Medical Record Review Study

Since 2015 an expert panel of clinicians, infectious disease (ID) specialists, anesthesiologists, surgeons, physicians, and hospital pharmacists have begun to manage hospital infection control and surveillance in two of the largest tertiary care hospitals of Southern Italy (AORN A. Cardarelli and Ospedali dei Colli) according to an antimicrobial stewardship program. This panel of experts was specifically involved in the evaluation of the efficacy and safety of antibiotics that were administered to patients admitted for disparate clinical conditions, including surgical and oncological morbidities, being at risk to develop infections.

A part of this project was dedicated to the assessment of the safety profile of fosfomycin. Specifically, a retrospective medical record review study was performed from June 2017 to June 2019 in order to assess the safety profile of fosfomycin in terms of the occurrence of hypernatremia cases. For this retrospective medical record review study, we included patients from two hospitals in Southern Italy. For each patient, the following information was collected: median age (interquartile range, IQR), sex, body mass index (BMI), smoking status, antibiotic treatment regimen and therapeutic indications, type of hospital stay (intensive care unit (ICU) or ward), hypernatremia severity classification (“Mild” with Na^2+^ ranged 145–155 mEq/ml, “Moderated” with Na^2+^ ranged 155–150 mEq/ml, and “Severe” with Na^2+^ over 160 mEq/ml), type of ADR, and drug withdrawal due to ADR. Patients were divided into two groups based on the occurrence of an ADR. Groups were compared by using the chi-square test and the Wilcoxon–Mann–Whitney test for categorical and continuous variables, respectively.

Moreover, to assess the impact of the training campaign (performed in June 2018) on the correct drug reconstitution in improving antibiotic safety, the number of hypernatremia cases was considered based on the periods 1 year before and after the training campaign (June 2017–2018 vs. June 2018–2019).

## Results

### Data From the EudraVigilance Database

**FIGURE 1:**
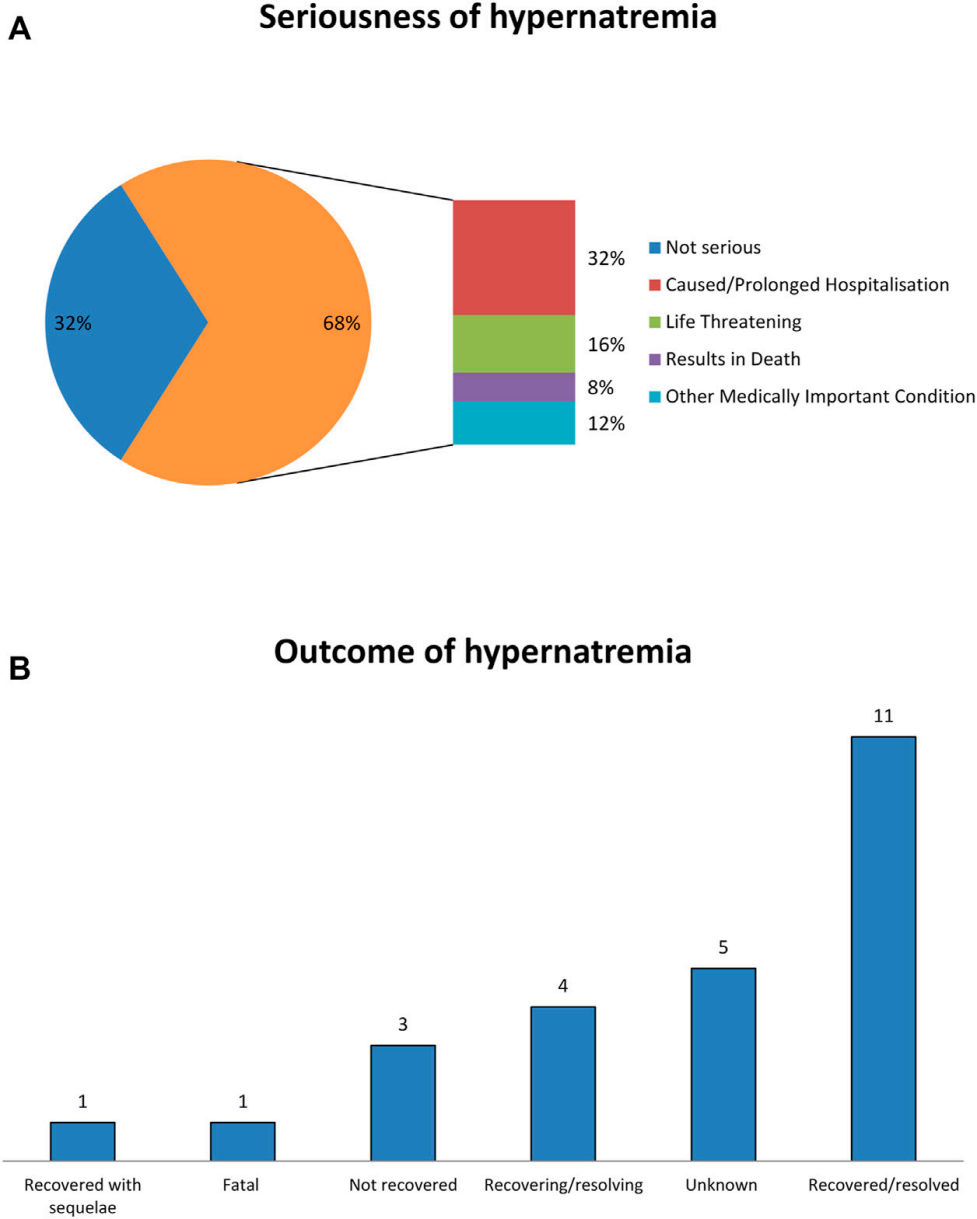
Seriousness criteria and outcomes of fosfomycin-induced hypernatremia from EudraVigilance data. **(A)** Seriousness of hypernatremia. **(B)** Outcomes of hypernatremia.

During the study period, 25 ICSRs with intravenous fosfomycin as suspected drug and hypernatremia as ADR were retrieved from the EV (Table 1). The distribution of patients who experienced fosfomycin-induced hypernatremia by age revealed that patients mainly belong to the age groups 65–85 years (52%) and 18–64 years (32%). No substantial difference was found for patients’ distribution by sex. Of all ICSRs, 68% were classified as serious. The outcome was defined as “recovered/resolved” in 44% of ICSRs and “recovering/resolving” in 16% of ICSRs. The outcome was not available for 20% of ICSRs, while it was indicated as “Fatal” in 4% of ICSRs (one case). Seriousness criteria and outcomes are shown in Figures 1A,B. In the majority of ICSRs, fosfomycin was the only suspected drug reported (72%), while in 48% of ICSRs, concomitant drugs were reported (Table 1). Regarding the primary source, all ICSRs were sent by healthcare professionals (data not shown).

**TABLE 1:** Demographic characteristics and distribution for seriousness, outcomes, primary source, number of suspected drugs other than fosfomycin, and number of concomitant drugs of ICSR related to hypernatremia associated with fosfomycin among those reported in the EudraVigilance database from the date of marketing authorization to 11 October 2021.

Variable	Level	All ICSRs (*n* = 25)
Age groups (%)	18–64 years	8 (32)
	65–85 years	13 (52)
	> 85 years	3 (12)
	Not specified	1 (4)
Sex (%)	Female	12 (48)
	Male	13 (52)
Seriousness (%)	Serious	17 (68)
	Not serious	8 (32)
Outcome (%)	Recovered/resolved	11 (44)
	Recovering/resolving	4 (16)
	Recovered with sequelae	1 (4)
	Not recovered/not resolved	3 (12)
	Fatal	1 (4)
	Unknown	5 (20)
Primary source (%)	Healthcare professional	25 (100)
Suspected drug(s) other than fosfomycin (%)	0	18 (72)
	1	4^a^ (16)
	3	3^b^ (12)
Concomitant drug(s) (%)	0	13 (52)
	1	2 (8)
	2	1 (4)
	>5	9 (36)

a Metronidazole, daptomycin, and ceftazidime.

b Levofloxacin, ambroxol hydrochloride, and ipratropium.

Fosfomycin was associated with an increased reporting frequency of hypernatremia (ROR 24.96; 95%CI 5.91–105.41; *p* value <0.001) when compared to gentamicin during the study period.

### Retrospective Medical Record Review Study

Overall, data related to 62 patients (48 men and 14 women) who had received fosfomycin were retrieved from the medical records. The treatment schedule included ceftolozane/tazobactam plus metronidazole plus fosfomycin or meropenem plus tigecycline plus fosfomycin or meropenem plus tigecycline plus colimicin plus fosfomycin or ceftazidime/avibactam plus tigecycline plus fosfomycin (Table 2). All drugs were used according to their approved therapeutic indications, and no off-label use has been identified (data not shown).

**TABLE 2:** Main clinical and demographic characteristics of patients who had received fosfomycin.

Variable	Level	Patients who experienced ADRs (*n* = 17)	Patients without ADRs (*n* = 45)	*p* value
Age, years	Median (IQR)	61 (58–66)	63 (60.5–72)	0.673
Sex	Female (%)	6 (35)	7 (16)	0.088
	Male (%)	11 (65)	38 (84)	
BMI	median (IQR)	23 (21.5–26)	24 (22–28)	0.934
Smoking status	n. (%)	4 (23)	8 (18)	0.609
Alcoholism	n. (%)	1 (6)	6 (13)	0.408
Treatment regimen	CT + MTZ + FOS	7 (41)	18 (40)	0.999
	CZA + TGC + FOS	3 (18)	8 (18)	
	MEM + TGC + FOS	5 (29)	13 (29)	
	MEM + TGC + COL + FOS	2 (12)	6 (13)	
Type of hospital stay	ICU (%)	7 (41)	16 (36)	0.683
	Ward (%)	10 (59)	29 (64)	
	Surgery	6 (35)	17 (38)	
	ICU	3 (18)	9 (20)	
	General medicine	1 (6)	3 (6)	
Hospital admission cause	Hepatectomy	5 (29)	14 (31)	0.999
	Cholecistectomy	3 (18)	9 (20)	
	CDP	3 (18)	8 (18)	
	Abdominal abscess	2 (12)	4 (8)	
	Total gastrectomy	2 (12)	4 (8)	
	Partial gastrectomy	1 (6)	3 (7)	
	Colon surgery	1 (6)	3 (7)	
Hypernatremia classification	Mild	5	-	—
	Moderate	4	-	
	Severe	2	-	

CT + MTZ + FOS, ceftolozane/tazobactam + metronidazole + fosfomycin; CZA + TGC + FOS, ceftazidime/avibactam + tigecycline + fosfomycin; MEM + TGC + FOS, meropenem + tigecycline + fosfomycin; MEM + TGC + COL + FOS, meropenem + tigecycline + colimicin + fosfomycin.

Regarding the safety profile, 17 out 62 patients (27.4%) experienced ADRs that were related to fosfomycin. Of these patients, 11 (7M/4F) experienced hypernatremia (Table 2), while the remaining patients experienced gastrointestinal signs and symptoms (nausea, vomiting, and diarrheal episodes, not *Clostridium* related) (data not shown). The main clinical and demographic characteristics of patients receiving fosfomycin and experiencing or not hypernatremia are shown in Table 2. No statistically significant difference was observed between patients with and without ADRs.

Furthermore, 63.7% of hypernatremia events were found throughout June 2017 up to June 2018, and according to clinical report evaluation and audit, they were related to the wrong reconstitution of the drug with saline solution and not glucose solution. According to these results, a surveillance and training campaign about the correct drug reconstitution and its monitoring was managed by the ID specialist and hospital pharmacist; however, despite the improvement in terms of the correct procedure for drug preparation, we still had, from June 2018 up to June 2019, four new hypernatremia cases in three males and one female over 60 years of age.

Drug withdrawal occurred in two patients for severe gastrointestinal ADRs and in one patient for lack of treatment efficacy to the antibiotic schedule ceftazidime/avibactam plus tigecycline plus fosfomycin. Regarding hypernatremia, a policy of close follow-up of patients with this electrolyte disorder was managed according to the ID specialist and hospital pharmacist, and therefore, only one patient required suspension.

## Discussion

In light of these data and literature evidence on antimicrobial stewardship too (Munez Rubio et al., 2019), antibiotics require an active follow-up from clinicians and hospital pharmacists not only to avoid an inappropriate use of these antibiotics and, therefore, the onset of resistance but also to minimize the risk of ADR. As we previously strongly suggested for antivirals (Nappi et al., 2019), a close collaboration among ID specialists, clinicians, and hospital pharmacists should be managed throughout the creation of a network that will allow greater monitoring of the safety profile of antibiotics.

From our retrospective medical record review study, we observed that 11 patients treated with fosfomycin experienced hypernatremia, while from the EV analysis, 25 ICSRs related to fosfomycin-induced hypernatremia were identified. Hypernatremia is generally induced by fluid and sodium imbalance. In critically ill patients, this adverse event can be multifactorial, but its development is the result of sodium overload or a loss of free water (Qian, 2019). Hypernatremia can also have an iatrogenic origin, especially in ill patients treated with fosfomycin. Indeed, fosfomycin is one of the most sodium-rich antibiotics that contained 0.33 g of sodium for each gram of IV fosfomycin. Therefore, hypernatremia is a common adverse event with IV fosfomycin, especially when it is given in high doses or for prolonged periods (Shorr et al., 2017). Indeed, to reduce the risk of wrong reconstitution and preparation of IV fosfomycin, a surveillance and training campaign was performed in our territory. However, after the campaign, four cases of hypernatremia were still reported, thus highlighting the need for further policy decision making.

Overall, our results showed that fosfomycin is a well-tolerated agent associated with a low frequency of suspension. Indeed, only four out of 62 patients suspended the treatment because of ADRs and only one patient because of hypernatremia. This result highlights that when good monitoring of the electrolyte disorder is performed and close collaboration is established among ID specialists and hospital pharmacists, we can have an optimization of the efficacy and safety of the therapy with fosfomycin. Indeed, close patient monitoring not only can avoid an unnecessary drug withdrawal but also reduce the burden associated with the development of severe hypernatremia-related complications such as the subarachnoid or subdural hemorrhage, which can cause permanent brain damage and major cardiovascular events (including fatal coronary heart disease, non-fatal myocardial infarction, and stroke) (Tsegka et al., 2021; Wannamethee et al., 2016). Moreover, the good safety profile of fosfomycin-related hypernatremia can be assumed by the low number of ICSRs (*n* = 25) that were retrieved from the European database EV.

Regarding the severity of hypernatremia, from the retrospective medical record review, we found that only one patient had severe hypernatremia, and from the EV analysis, we found that most cases were reported as serious. In this regard, it should be worth noting that the classification of seriousness in pharmacovigilance follows different criteria from those used in the clinical setting to establish the severity of hypernatremia. In the evaluation of seriousness, we should also consider the reporting bias of serious ADRs due to mandatory reporting obligations and that healthcare professionals are more prone to report serious ADRs. Indeed, the primary source for the reporting of ADRs was the healthcare professional in all ICSRs. Finally, according to our results, literature clinical data classified fosfomycin-induced hypernatremia in most cases as mild (Tsegka et al., 2021). From the disproportionality analysis, we found that fosfomycin was associated with a 25-times higher reporting frequency of hypernatremia compared to gentamicin. Indeed, according to the literature, gentamicin is associated with the occurrence of electrolyte and acid-base disorders, mainly due to the increase in the excretion of sodium and magnesium (Scoglio et al., 2021). Therefore, this antibiotic can exert opposite effects in regulating sodium levels.

Further active trial and audit are mandatory to improve the use of antibiotics in terms of preparation, administration, and monitoring in antimicrobial stewardship programs. In conclusion, intravenous fosfomycin, despite its efficacy and relative safety, may need a more careful follow-up by clinicians and hospital pharmacists, particularly in those wards not familiar with antibiotics use. Indeed, the lack of close surveillance of hypernatremia may result in an increase in healthcare costs, not only due to prolonged hospitalization but also for possible complications related to unnecessary drug withdrawal.

## Study Limitations

This is a retrospective analysis of medical records related to patients who experienced hypernatremia after having received fosfomycin. The limited number of patients included in the study (*n* = 62) together with the absence of a control group did not allow us to draw firm conclusions. Indeed, because of the nature of the study, we could not rule out the presence of other confounding variables that might have affected our results. Larger sample-sized and controlled studies are required in order to better assess the safety profile of intravenous fosfomycin.

The second part of our study was based on data from the European spontaneous reporting system. In this regard, it is well known that all spontaneous reporting systems carry some intrinsic limitations, such as under-reporting. Indeed, we found analyzed data related to only 25 ICSRs reporting the association between fosfomycin/hypernatremia. Another important limitation of such systems is represented by the poor quality of information listed in each ICSR. As we used EV data obtained from the EMA website (www.adrreports.eu), we cannot exclude that important clinical data were not listed in evaluated ICSRs. Therefore, the effects of concomitant illnesses or therapy cannot be fully excluded in this analysis. Thus, we are aware that the real safety profile of fosfomycin in terms of hypernatremia occurrence needs to be confirmed by the results obtained from ad hoc studies.

## Conclusion

We carried out two separate studies, both in real-life settings, with the aim to evaluate the fosfomycin-induced hypernatremia. Our results primarily showed that fosfomycin is safe and associated with a low frequency of suspension due to ADRs. Indeed, only four patients suspended the treatment due to ADRs. Moreover, this study demonstrated that surveillance and training campaigns on the correct drug preparation need to be strengthened to allow a reduction of hypernatremia cases, considering that most of our cases were related to reconstitution errors. Moreover, our results demonstrated that when tight monitoring and management of the event is performed with a close collaboration between the ID specialist and the hospital pharmacist there, is a reduction in antibiotic withdrawal. Given the clinical significance of high sodium content and its potential fatal clinical consequences, the parenteral use of fosfomycin should be carefully monitored, especially in patients with underlying heart disease.

## Data Availability

The pharmacovigilance datasets are readily available. This data can be found at www.adrreport.eu. The medical record datasets are not readily available because of institutional policy restrictions. Requests for these datasets should be directed to annamaria.mascolo@unicampania.it.
